# Reply to Comment on Watanabe, A.; Kadota, Y.; Yokoyama, H.; Tsuruda, S.; Kamio, R.; Tochio, T.; Shimomura, Y.; Kitaura, Y. Experimental Determination of the Threshold Dose for Bifidogenic Activity of Dietary 1-Kestose in Rats. *Foods* 2020, *9*, 4

**DOI:** 10.3390/foods9040527

**Published:** 2020-04-22

**Authors:** Ayako Watanabe, Yoshihiro Kadota, Takumi Tochio, Yoshiharu Shimomura, Yasuyuki Kitaura

**Affiliations:** 1 Laboratory of Nutritional Biochemistry, Department of Applied Biosciences, Graduate School of Bioagricultural Sciences, Nagoya University, Nagoya, Aichi 464-8601, Japan; watanabe.ayako@i.mbox.nagoya-u.ac.jp; 2 B Food Science Co., Ltd., Chita, Aichi 478-0046, Japan; y-kadota@bfsci.co.jp (Y.K.); t-tochio@bfsci.co.jp (T.T.); 3 Department of Food and Nutritional Sciences, College of Bioscience and Biotechnology, Chubu University, Kasugai, Aichi 487-8501, Japan; shimo@agr.nagoya-u.ac.jp

The manuscript entitled “Comment on Experimental Determination of the Threshold Dose for Bifidogenic Activity of Dietary 1-Kestose in Rats” by Shen et al. gives comments on our published work [1], particularly regarding the primers used to detect *Bifidobacterium*. Although Shen et al. raise questions on primer specificity and the qPCR method we used, these primers have been used for qPCR (using SYBR green as the fluorescent dye) in many other papers [2,3,4,5,6,7,8,9,10,11,12,13,14,15,16,17,18,19,20,21,22,23]. *Gardnerella* spp., *Scardovia inopinata,* and *Parascardovia denticolens* are closely related to *Bifidobacterium* spp., and could be theoretically amplified, but these former species have been found only in genitals, urethra, and caries of human, and have not been detected in cecum contents of rats by metagenomic analysis using the EzBioCloud database (our unpublished data). Furthermore, although microbes with more than one mismatch in the primers might be amplified, such data have not been shown.

qPCR was performed in our study as follows. Frozen cecum content samples were thawed on ice, and approximately 100 mg of each sample was suspended in 4 M guanidium thiocyanate, 100 mM Tris-HCl (pH 9.0), and 40 mM EDTA. Samples were then homogenized with zirconia beads using the FastPrep FP100A instrument (MP Biomedicals, Santa Ana, CA, USA). DNA was extracted from bead-treated suspensions using magLEAD 12gC and MagDEA Dx SV (Precision System Science, Matsudo, Japan). DNA concentrations were estimated by spectrophotometry using the ND-1000 spectrophotometer (NanoDrop Technologies, Wilmington, DE, USA), and final DNA concentrations ranged from 26.2 to 74.9 ng/μL. We used a diluted genomic DNA solution (from 5 to 10 times) as a template and SYBR green as a fluorescent dye during qPCR. The data were shown as the copy number per 1 g of cecum content as calculated based on the weight of the cecum content used for genomic DNA extraction, dilution rate, and Ct values (between 18 and 24 in duplicate).

Shen et al. also show the bifidogenic activities of fructo-oligosaccharides fructo-oligosaccharides (FOSs) in humans as well as in vitro and suggest that the equivalence of 1-kestose in foodstuffs for humans should be calculated by the sensitivity index (6.25) between humans and rats used in toxicological studies. As we described in our paper, further studies are required to determine the minimum effective dose of 1-kestose in human patients with conditions such as obesity and type 2 diabetes.
